# Sudden cardiac arrest during endovascular embolization of carotid artery aneurysm: A case report and literature review

**DOI:** 10.1097/MD.0000000000036888

**Published:** 2024-01-26

**Authors:** Ziqiang Liu, Xuecai Ma, Tianhui Yang

**Affiliations:** a Department of Anesthesiology, Weihai Municipal Hospital, Waihai, China; b Department of Anesthesia Surgery, Wuming Hospital Affiliated to Guangxi Medical University, Nanning, China.

**Keywords:** cardiopulmonary resuscitation, carotid artery aneurysm, interventional embolization, sudden cardiac arrest

## Abstract

**Rationale:**

Endovascular embolization has been widely applied in carotid artery aneurysm due to less trauma and simpler procedures than open surgery. Sudden cardiac arrest is a rare event that may cause severe consequences during endovascular embolization. Risk factors of perioperative cardiac arrest include cardiac surgery, younger age, comorbid conditions, and emergency surgery.

**Patient concerns:**

A 62-year-old male patient had hypertension for 15 years and experienced sudden cardiac arrest of pulseless electrical activity during endovascular embolization.

**Diagnoses:**

He was diagnosed with a 3.5 × 2.5 mm aneurysm.

**Interventions:**

Chest compression and effective interventions were given.

**Outcomes:**

He was resuscitated by cardiopulmonary resuscitation and systematic therapy.

**Lessons:**

This case may provide experience in the management of sudden cardiac arrest during endovascular embolization of a carotid artery aneurysm.

## 1. Introduction

Annually, over 200,000 patients suffer from in-hospital cardiac arrest in U.S.^[1]^ Cardiopulmonary resuscitation and defibrillation play a critical role in prolonging patient survival.^[2]^ Sudden cardiac arrest is a leading cause of death. In Europe, annual incidence of 119 per 100,000 adults has been reported.^[3]^

However, sudden cardiac arrest during endovascular embolization has been rarely reported. We presented a 62-year-old male patient with a 3.5 × 2.5 mm aneurysm of the left internal carotid artery, who experienced pulseless electrical activity. As a subtype of sudden cardiac arrest, pulseless electrical activity is characterized by unresponsiveness and impalpable pulse under sufficient electrical discharge, aiming to provide reference for sudden cardiac arrest during endovascular embolization.

## 2. Case report

### 2.1. Baseline data

A 62-year-old male patient was diagnosed with a carotid aneurysm, 3.5 × 2.5 mm, at the end of the cavernous sinus segment of the left posterior internal carotid artery. Endovascular embolization under general anesthesia was adopted. He had hypertension for 15 years, received valsartan for 2 years, and blood pressure was 130/80 mm Hg. He underwent carotid endarterectomy for the left internal carotid artery stenosis (lumen stenosis >95%) 1 year ago. Written informed consent was obtained from the patient.

### 2.2. Anesthesia procedures

In operation room, blood pressure was 160/80 mm Hg and heart rate was 90 beats/min. Intravenous midazolam 2 mg, sufentanil 30 µg, etomidate 40 mg, vecuronium 8 mg, and penehyclidine 0.5 mg were applied for anesthesia induction, and No. 7.5 reinforced endotracheal tube was inserted. Ventilator settings were volume control mode, tidal volume was 5 mL/kg. Invasive arterial pressure was monitored after general anesthesia. After anesthesia, blood pressure declined to 80/40 mm Hg, and fluid replacement and intravenous infusion of norepinephrine 0.1 mg/h were given to elevate blood pressure to 110–130/50–70 mm Hg, and heart rate was 60 to 70 beats/min. General anesthesia was maintained with 2% sevoflurane.

### 2.3. Surgical procedures

The left femoral artery was punctured by Seldinger technique, 6F arterial sheath was inserted, and whole body was heparinized. Under the guidance of Roadmap, 6F coaxial guiding catheter was selected to the end of vertical segment of petrous bone of the left internal carotid artery by 0.035-inch loach guide wire, and three dimensional imaging was performed to identify the aneurysm. The front end of a Headway-21 microcatheter was molded into a single bend shape, and placed to M1 segment of the left middle cerebral artery guided by 0.014-inch guide wire, and Headway-17 microcatheter was molded. During release process, spring coil was unstable, and 4.5 × 20 mm livis stent was released along Headway-21 microcatheter, and the midpoint of the stent was located at the neck of aneurysm, and the stent was expanded ideally. Angiography showed that aneurysm embolization was successful. During angiography, blood pressure dropped from 110/50 to 40/40–20/20 mmHg, and direct arterial pressure waveform was absent. Angiography indicated that blood flow rate was extremely slow. Electrocardiogram (ECG) was normal and heart rate was 60 beats/min. The anesthesiologist immediately carried out chest compression. The waveform of direct arterial pressure fluctuated regularly after chest compression. Then, the waveform of direct arterial pressure disappeared. The possibility of sudden cardiac arrest was considered. One hundred micrograms of norepinephrine was given intravenously, whereas blood pressure was not improved. Chest compression was immediately and persistently delivered and direct arterial pressure was maintained at 130–170/60–90 mm Hg. After 10-minute chest compression, blood pressure was 60–80/30–40 mm Hg. After intravenous injection of 1 mg epinephrine, arterial pressure was increased to 299/230 mm Hg. Sharp blood pressure fluctuation endured for 5 minutes, and arterial pressure was 180/80 mm Hg. ECG showed significant ST-segment elevation complicated frequent premature ventricular beats. After 20-minute emergency rescue, ECG morphology was restored to preoperative level, arterial pulse was stable, and he was successfully rescued. ECG showed interventricular septum was thickened by 18 to 20 mm, and no significant myocardial ischemic changes. Continuous inhalation of sevoflurane was given to maintain blood pressure at 140/70 mm Hg. He was transferred to intensive care unit. Bilateral pupils were 1 to 2 mm, and no obvious light reflection. Multidisciplinary team (Department of Cardiology, ECG, and intensive care unit) immediately delivered emergency rescue. Blood gas analysis, ECG, and color Doppler echocardiography yielded normal results. At postoperative 2 days, he was extubated and transferred to routine ward, discharged after recovery.

## 3. Discussion

The causes of intraoperative sudden cardiac arrest consist of hyperkalemia, excessive excitement of vagus nerve, and massive blood loss.^[4]^ In this case, possible causes of intraoperative sudden cardiac arrest are summarized. First, the patient stayed up all night before operation, and sympathetic nerve was tired and vagus nerve excitability was increased intraoperatively. Second, intraoperative carotid sinus reflex (CSR) triggering provoked excessive excitement of vagus nerve and significant blood pressure fluctuations. Third, long-term dehydrated treatment before operation caused insufficient blood volume. As Goto et al^[5]^ described, CSR is a rare complication of pipeline embolization device deployment. They examined CSR triggering during pipeline embolization device deployment in 37 patients. One patient had transient cardiac arrest and 2 had severe transient bradycardia. Onodera et al^[6]^ also reported a case of 30-second cardiac arrest during carotid body tumor resection due to CSR in a 20-year-old male patient. Chest compression was initiated, atropine 0.5 mg was administered. Surgery was resumed after placing a temporary pacemaker through the left subclavian vein. Therefore, the findings indicate the significance of pacemaker placement before carotid body tumor resection.

According to 2015 American Heart Association guidelines update for cardiopulmonary resuscitation and emergency cardiovascular care,^[7]^ chest compression is the key procedure of cardiopulmonary resuscitation.

For patients with cardiac arrest unresponsive to cardiopulmonary resuscitation, extracorporeal life support can assist cardiac resuscitation.^[8]^ Fagnoul et al^[9]^ have proposed that hypothermia protection is an important approach for patients with cardiac arrest.

Brahmajee et al^[10]^ qualitatively analyzed 4 essential qualities of emergency rescue including team design, team composition and roles, communication and leadership during cardiac arrest, and training and education. Resuscitation teams at top-performing hospitals demonstrate the features including dedicated or designated resuscitation teams, multidisciplinary participation as team members, clear roles and responsibilities, smooth communication and leadership, and in-depth mock codes.

In the present case, clinical experience can be summarized. First, invasive direct arterial pressure monitoring is delivered. Early detection of invalid cardiac ejection and effective guidance of rescue measures are conducted, such as chest compressions. Second, intraoperative systemic heparinization is given to prevent disseminated intravascular coagulation. Third, breathing is controlled by anesthesia machine during general anesthesia, and intraoperative inhaled oxygen concentration can be 100%. Short-term cardiac arrest will not cause hypoxia in vital organs. Fourth, ECG, oxygen saturation, direct arterial pressure, and end-tidal carbon dioxide partial pressure can be adopted. After emergency rescue, patients should be examined by color Doppler ultrasound to guide advanced life support. Fifth, patients should wear ice cap and the neck is subject to cold compression.

## 4. Study limitations

First, ECG is not performed prior to cardiopulmonary resuscitation. Second, use of adrenaline may cause hemodynamics instability and increase the risk of cerebral hemorrhage and heart failure. For patients with electromechanical dissociation subtype of sudden cardiac arrest, low-dose adrenaline should be administered. The findings remain to be validated in larger sample size investigations.

## 5. Conclusion

Cardiopulmonary resuscitation is priority for emergency management of intraoperative sudden cardiac arrest. Intraoperatively, patients should be whole-body heparinized to guarantee that sudden cardiac arrest will not cause disseminated intravascular coagulation. Chest compression should be immediately taken. Low-dose adrenaline should be administered.

## Author contributions

**Conceptualization:** Ziqiang Liu, Xuecai Ma, Tianhui Yang.

**Data curation:** Ziqiang Liu, Xuecai Ma, Tianhui Yang.

**Investigation:** Ziqiang Liu, Xuecai Ma, Tianhui Yang.

**Methodology:** Ziqiang Liu, Xuecai Ma, Tianhui Yang.

**Writing – original draft:** Ziqiang Liu, Xuecai Ma, Tianhui Yang.

**Writing – review & editing:** Ziqiang Liu, Xuecai Ma, Tianhui Yang.
